# Distinct Differences in Chromatin Structure at Subtelomeric X and Y' Elements in Budding Yeast

**DOI:** 10.1371/journal.pone.0006363

**Published:** 2009-07-23

**Authors:** Xuefeng Zhu, Claes M. Gustafsson

**Affiliations:** 1 Department of Biochemistry and Cell Biology, University of Gothenburg, Gothenburg, Sweden; 2 Max-Planck-Institut für Biologie des Alterns, Cologne, Germany; University of Munich and Center of Integrated Protein Science, Germany

## Abstract

In *Saccharomyces cerevisiae*, all ends of telomeric DNA contain telomeric repeats of (TG_1–3_), but the number and position of subtelomeric X and Y' repeat elements vary. Using chromatin immunoprecipitation and genome-wide analyses, we here demonstrate that the subtelomeric X and Y' elements have distinct structural and functional properties. Y' elements are transcriptionally active and highly enriched in nucleosomes, whereas X elements are repressed and devoid of nucleosomes. In contrast to X elements, the Y' elements also lack the classical hallmarks of heterochromatin, such as high Sir3 and Rap1 occupancy as well as low levels of histone H4 lysine 16 acetylation. Our analyses suggest that the presence of X and Y' elements govern chromatin structure and transcription activity at individual chromosome ends.

## Introduction

In *Saccharomyces cerevisiae*, telomeric DNA consists of tandem repeats of (TG_1–3_)*n* [1]. The length of these repeats varies between individual chromosomes and strains, but the average is typically about 300 nucleotides. In addition, two subtelomeric repeat elements called Y' and X are often found associated with the telomeric repeats [2]. The Y' element is located next to the telomeric repeats at many but not all telomeres, and is present either as a single copy or as a tandem repeat of two to four copies. The function of Y' elements are not known, but the structure and distribution of these sequences, are consistent with an origin as a mobile genetic element, even if the Y' elements today only moves via recombination [3]. The structure of X elements is more variable, ranging in size from 300 bp to 3 kb, but each X element contains a “core-X” repeat that is found at nearly all telomeres. Based on the distribution of these structural elements, the budding yeast chromosome ends can be divided into X and X-Y' types.

Genes placed near telomeres are transcriptionally repressed, which is a phenomenon termed the telomere position effect (TPE) [4]. TPE was discovered by placing a reporter gene immediately adjacent to the telomeric TG_1–3_ tract, thereby generating a telomere that lacked both X and Y' elements [5]. TPE is also observed at native yeast telomeres, but the level of TPE can vary substantially from telomere to telomere, even in the same strain background [6]. At some chromosome ends, telomere-adjacent genes are repressed in a small percentage and at others in 100% of the cells. Hence, telomeres do not only have different subtelomeric structures, but they also exhibit different levels of TPE. Mutations analysis suggest that X elements may contribute to TPE [7]. In some organisms, heterochromatin near telomeres can even contain active genes. For instance, the *rolled* gene in Drosophila is located in heterochromatin and its expression is essential for viability [8]. Another interesting phenomenon is transcription of telomeric repeats found in fission yeast and human [9], [10]. This telomeric non-coding RNA can block the human telomerase activity *in vitro* [11].

In budding yeast, many proteins have been identified, which can modulate TPE [12]. Prominent among these are the Sir complex proteins (Sir2, Sir3, and Sir4), the Ku heterodimer (Ku70 and Ku80), and Rap1, a sequence-specific telomeric DNA binding protein, which spread into subtelomeric regions. Rap1 and Ku have been show to bind telomere repeat DNA and subsequently recruit the Sir proteins [13]. The Rap1/Ku/Sir complex propagates towards the subtelomeres via interactions between the Sir proteins and histone tails [14]. Sir2 has a histone deacetylase activity, which deacetylate lysine 16 on histone H4 (H4K16) and enables Sir3 and Sir4 to bind the hypoacetylated tails [15].

In this study, we compare the histone density and nucleosome distribution at X and Y' elements. Unexpectedly, we find that X elements lack histones and a defined nucleosome structure. In contrast, Y' elements display high nucleosome density. Furthermore, at telomeres only containing X elements, we fail to observe significant H4K16Ac levels. In contrast, we do observe, significant levels of H4K16Ac at some regions in Y' elements and transcription analysis reveals that Y' elements are transcriptionally active. The Sir3 protein binds to X elements, but not Y' elements, and deletion of the *SIR3* gene leads to derepression of X element transcription. Our results reveal that X and Y' elements in budding yeast have distinct chromatin structure and this variation may help to explain the variations in TPE seen at different telomeres.

## Results

### Histone distributions in subtelomeric regions

**Figure 1:**
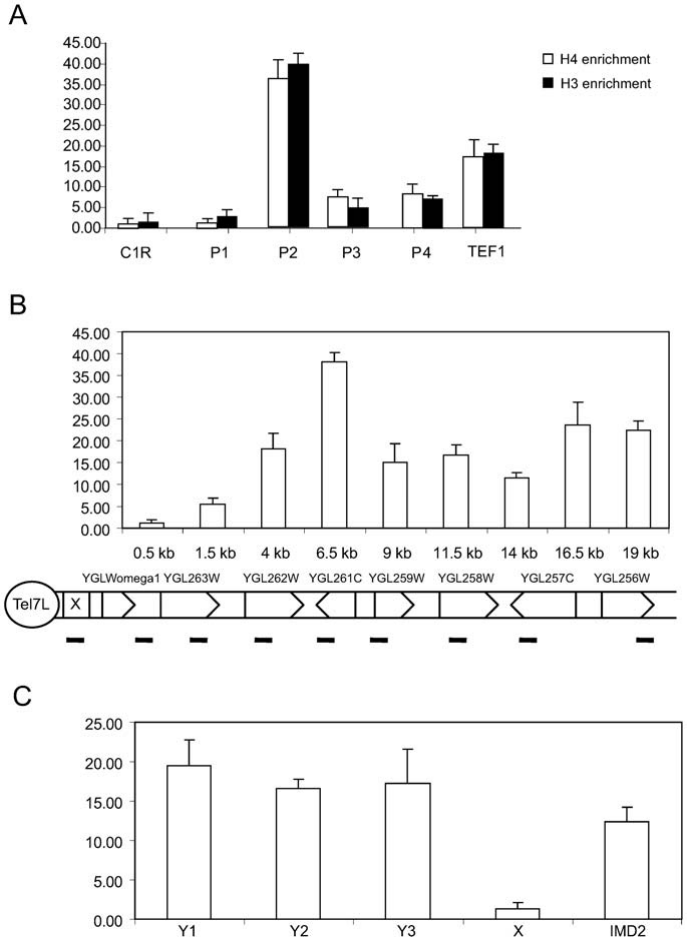
ChIP analysis of histone H3 and H4 occupancy at chromosome ends. A. Precipitated DNA was quantified by real-time PCR using primer pairs amplifying X elements (C1R, P1, P3, and P4) or Y' elements (P2). A primer pair amplifying the *TEF1* coding region was used as a euchromatin control. Error bars show standard deviations. B. Histone H3 occupancy at Tel7L, which contains an X element, but no Y' element. Precipitated DNA was quantified by real-time PCR using primer pairs covering the indicated positions. Error bars show standard deviations. C. High histone H3 occupancy at different positions on the Y' element. As a control we measured H3 occupancy at X elements and at the subtelomeric *IMD2* gene. The analysis was as described for panel B.

We first measured histone H3 and H4 occupancy at chromosome ends. We used 5 different primer pairs. Due to sequence similarities between repeat elements present at different chromosome ends, we could only find one unique primer pair, which was specific to the X element at telomere C1R. The other primer pairs amplified multiple X elements. The P1, P3, and P4 primer pairs amplified between 3 to 12 different X elements, whereas the primer pair P2 amplified 13 different Y' elements (Supplementary table S1). Primer pairs corresponding to X elements detected low levels of histone occupancy, whereas the primers detecting Y' elements displayed high histone occupancy (Figure 1A). For comparison, the coding region of the *TEF1* gene was used as an example of euchromatin histone occupancy. Our analysis showed that the occupancy at Y' elements was even higher than the histone occupancy in the euchromatic *TEF1* coding region.

We next chose to analyze the C7L chromosome end in more detail. This region contains an X only structure. We designed primers, which covered large regions of the chromosome end and monitored histone occupancy (Figure 1B). All primer pairs were specific, except that for the X element, which also recognized similar elements in other chromosomes. At C7L, we observed low levels of histones over the X element situated near the TG_1–3_ repeats and increasing levels of histones at positions at a distance of 1.5, 4, and 6 kb from the chromosome end.

We next analyzed histone occupancy at different positions in Y' elements (Figure 1C). As previously, the primers detected Y' elements present at multiple chromosome ends (for details see supplementary table S1). The histone density remained high over the entire Y' element as measured by primer pairs situated at 0.8 kb, 1.8 kb, and 3.0 kb from the chromosome end. As controls we used a primer pair detecting X elements and another primer detecting the coding region of the *IMD2* gene located 1.3 kb from the X-element in the C8R chromosome end. As previously, X elements contained low levels of H3, whereas the coding region of *IMD2* contained H3 levels similar to Y' elements.

**Figure 2:**
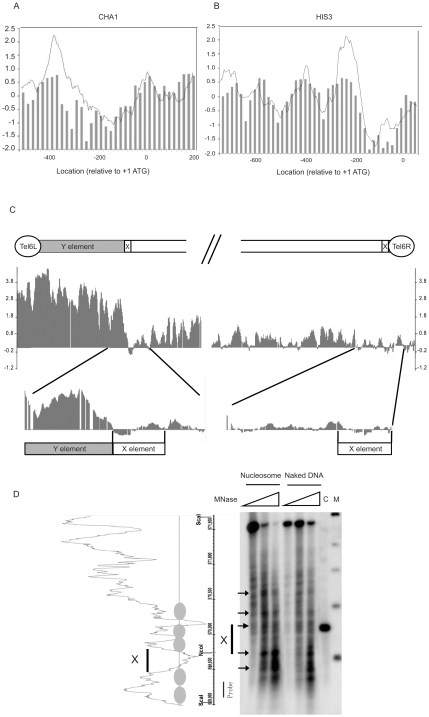
High resolution profiling of nucleosome positioning near telomeres. A and B. A comparison between our tilling array data and previously reported nucleosome positions. The Y axis is log2 and each bar represents enrichment in the immunoprecipitated nucleosome fraction relative to a naked DNA control sample. The X axis represents the distance to ATG. Bars present data from Yuan et al., 2005, which corresponds to median normalized ChIP-chip values for nucleosome occupancy. The grey traces are our nucleosome positioning data based on DNA microarray analysis of the indicated regions. Panel A shows the *CHA1* promoter and Panel B shows the *HIS3* promoter. C. High nucleosome occupancy was seen on the Y' element of Tel6L, whereas low occupancy was seen on the X elements of both Tel6L and Tel6R. D. Micrococcal nuclease mapping of nucleosomes at the right end of chromosome V. The left panel indicates the nucleosome distribution from tiling array data. The position ruler indicates the *Sca* I and *Nco* I sites as well as the location of the radioactive probe used for Southern hybridization. The right panel demonstrates micrococcal nuclease mapping data for the corresponding region. Microcoocal nuclease (0, 2, and 4 units) were used for digestion of nucleosomal DNA and the naked DNA control. Lane C contains genomic DNA digested by *Sca* I and *Nco* I, to localize the X-element region. Lane M contains a DNA size marker (500 bp, 1 kb, 1.5 kb, 2 kb, and 3 kb). The black bar indicates the location of the X element region.

Our observations so far suggested that histones are absent or at least very scarce on X elements. To further address this possibility, we analyzed positioning of nucleosomes over telomeres using DNA microarray technology. We isolated 146-bp mononuclesomes from micrococcal nuclease digested chromatin. The purified 146-bp mononucleosomal DNA was hybridized to a *S. cerevisiae* tiling array (Affymetrix). Naked genomic DNA randomly digested with micrococcal nuclease was also hybridized to tiling arrays and used to define the background. The data were normalized with the Affymetrix Tiling Array software (TAS) and visualized with the Integrated Genome Browser (IGB). To validate nucleosome maps generated this way, we compared our findings with previously published data for the *CHA1* and *HIS3* promoter regions [16]. This comparison showed a strong concordance and demonstrated that our nucleosome distribution is similar to that found by others (Figure 2A and B).

We next analyzed the nucleosome occupancy at X and Y' elements. Figure 2C displays the nucleosomal organization of the left and right ends of chromosome VI. The left end has a X-Y' organization, whereas the right end has only an X element. On the left end, nucleosome density is significantly higher than the genome wide average level and nucleosomes cover the entire Y' element. Consistent with our histone ChIP results, nucleosomes are almost completely absent from the X elements, regardless if the X element is located at the extreme telomere end (right end) or after an adjacent Y' element (left end). The same pattern was evident at all X-Y' type chromosome ends analyzed (Supplementary information, Figure S1 A – D).

Our results therefore demonstrate that X and Y' elements differ in histone as well as nucleosome density. To further confirm that X elements lack a nuclesomal organization, we used micrococcal nuclease mapping and indirect labeling to map nucleosome positioning at the right end of chromosome V. The nuclease mapping results supported our tiling array data, since no stably positioned nucleosomes were observed on the X element region.

### Rap1 and Sir3 bind to X elements

**Figure 3:**
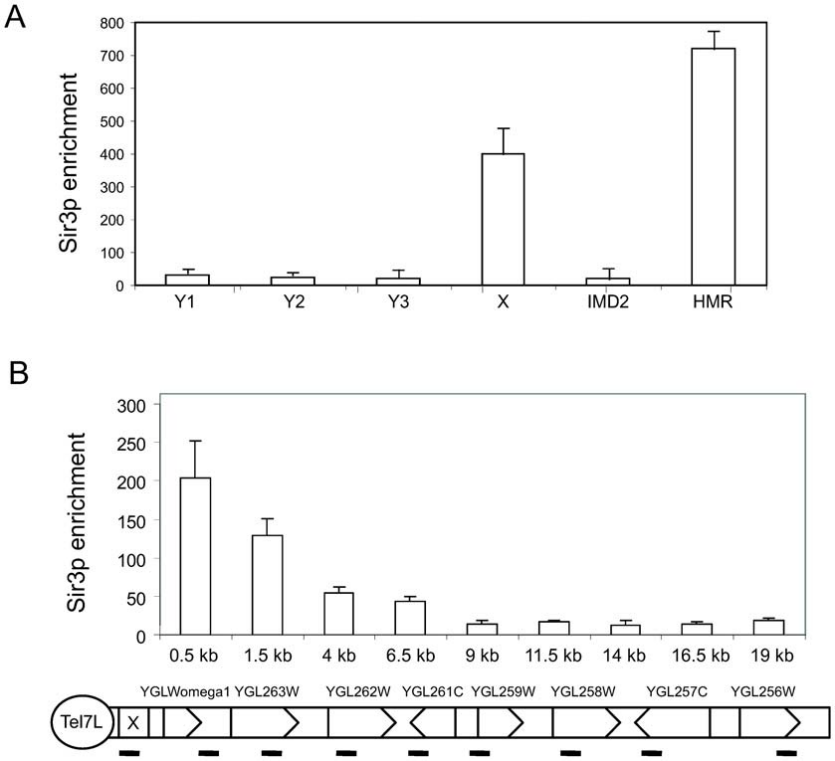
The Sir3 protein is enriched on X elements. A. ChIP analyses of Sir3 occupancy. Precipitated DNA was quantified by real-time PCR using primer pairs covering the indicated positions. HMR E primers were used as a positive control. Error bars show standard deviations. B. The same analysis as in Panel A, but on Tel7L, which contains an X, but not a Y' element.

Since stably positioned nucleosomes are absent from X elements, we wondered if the heterochromatin associated proteins Rap1 and Sir3 could bind these regions. We first analyzed Sir3 occupancy using the same set of primer pairs as in figure 1C. Our analyses revealed that the Sir3 protein was highly enriched at X elements, but we could not observe significant Sir3 occupancy at three different locations investigated in Y' elements (Figure 3A). As a positive control, we monitored Sir3 occupancy using a primer pair amplifying the *HMR* locus [17].

We next analyzed Sir3 occupancy at the C7L chromosome end using the same primer pairs as in figure 1B. We observed high Sir3 occupancy over the X element situated near the TG_1–3_ repeats and gradually decreasing levels of Sir3 towards the euchromatic regions of the chromosome (Figure 3B).

Genome-wide profiles for Rap1 binding have been reported by several different laboratories [18], [19], [20], but the Rap1 occupancy at X elements was not specifically analyzed. We reanalyzed these previously published Rap1 binding data [18] to investigate if Rap1 also binds to X elements. In excellent agreement with our Sir3 binding data, we found that Rap1 is highly enriched at X elements, but largely absent from the Y' element regions (Supplementary figure S2 A and B).

### Histone modifications at Y' elements

**Figure 4:**
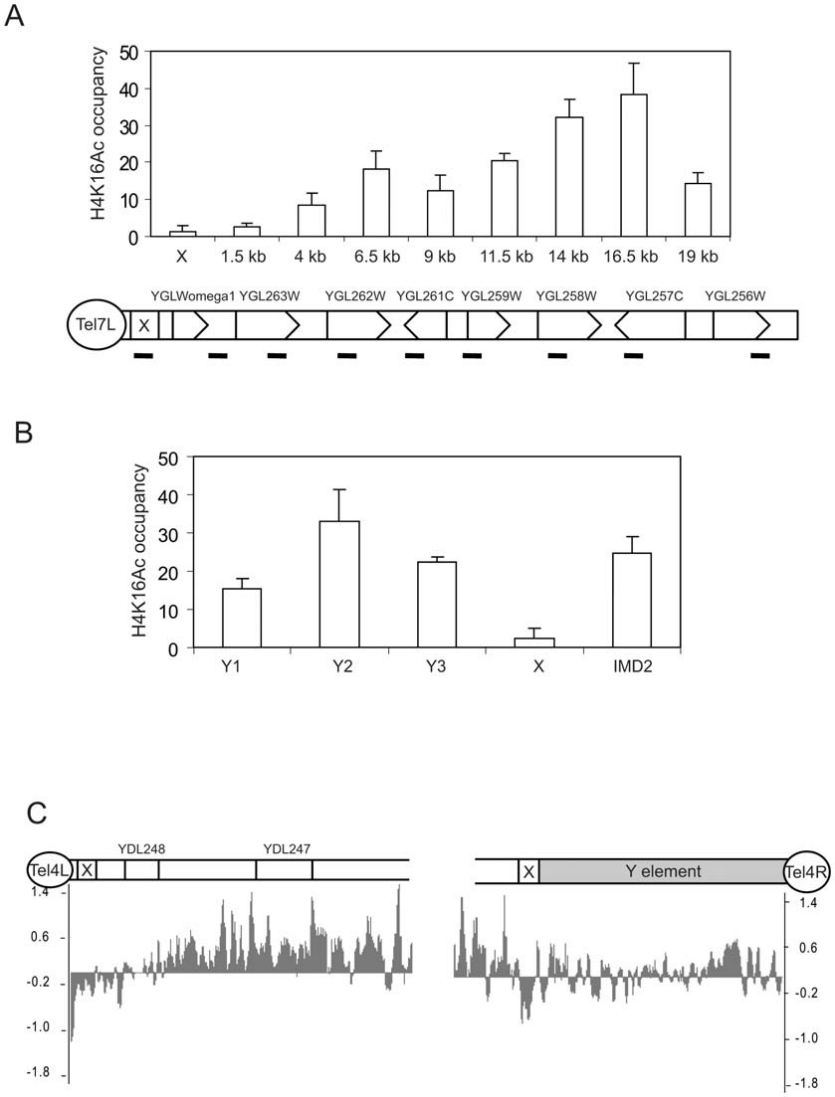
The H4K16Ac modification can be identified on Y', but not on X elements. A. ChIP analyses of H4K16Ac occupancy on Tel7L, which contains an X, but no Y' element. Precipitated DNA was quantified by real-time PCR using primer pairs covering the indicated positions. Error bars show standard deviations. B. ChIP analyses of H4K16Ac as in panel A, but analyzing different positions on Y' elements. C. High resolution map H4K16Ac using a DNA tiling array (Affymetrix) reveal that H4K16Ac is present on the Y' element, but absent from the X elements of chromosome 4 telomeres.

The Sir2 protein acts as a deacetylase and telomere regions contain hypoacetylated H4K16 due to spreading of the Sir2/Sir3/Sir4 complex. Since we had seen Sir3 binding to X elements, we decided to compare H4K16Ac levels between X only and X-Y' type chromosome ends, using the same primer pairs as in figures 1 and 3. In agreement with Sir3 binding data, there were low levels of H4K16Ac at the X elements (Figures 4A and 4B). Interestingly, the levels of H4K16Ac in the Y' elements, were much higher than that in X elements and similar to what was seen in the *IMD2* coding region (Figure 4B and data not shown). To further confirm our observations that H4K16 remains acetylated on Y' elements, we mapped genome wide H4K16Ac patterns using tiling arrays (Figure 4C and Supplementary information Figure S3 A – D). The analysis confirmed our ChIP results and revealed H4K16Ac at Y' elements, but low H4K16Ac on the X elements. The length of the hypoacetylated region varied between different chromosome ends, likely reflecting the spreading distance of heterochromatin.

### Transcription at Y' elements

**Figure 5:**
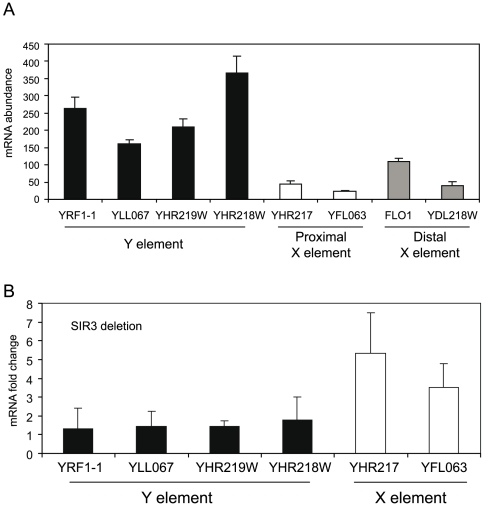
Y' elements are transcriptionally active, whereas X elements are repressed. A. mRNA quantification of different subtelomeric ORFs. Randomly selected genes located in X and Y' elements are displayed. Error bars show standard deviations. B. Deletion of *SIR3* leads to derepression of X element genes, but does not affect genes located to Y' elements. The fold change of transcription in wt and *sir3* cells are indicated. Error bars show standard deviations.

X and Y' elements contain some short open reading frames (ORFs), even if most of these correspond to pseudogenes [21]. To compare the transcription levels in different telomeric regions, we picked 4 primer pairs detecting ORFs in Y' elements, 2 primer pairs detecting X elements, and primer pairs detecting 2 telomere proximal genes. We performed reverse transcription followed by real time quantitative PCR (RT-QPCR) analyses. To quantity the transcript abundance, 4 genes with known abundance were chosen as standards (http://downloads.yeastgenome.org/). Interestingly, primers detecting Y' element sequences showed 5–10 fold higher transcription than primers detecting X elements or genes in a telomere proximal position (Figure 5A). In agreement with these observations, high transcription activity has previously been described for a reporter gene in a Y' element, suggesting that Y elements are not subject to transcription silencing [22].

### Deletion of the *SIR3* gene leads to derepression of X elements

Our results showed that the Sir3 protein interacts with X elements. We examined changes in gene transcription in a *sir3* deletion strain, in order to analyze the requirement of Sir proteins for transcription silencing of genes located in X or Y' elements. RT-QPCR results only revealed derepression of genes located in X elements, whereas the Y' element located genes remained unchanged (Figure 5B). Genome-wide transcription changes in a *sir3* deletion strain has been published before [23] and careful reanalysis of these data also revealed that deletion of *sir3* causes a depression of genes located to X elements, but not Y' elements, nicely correlating with our previous observation that Sir3 is enriched at X elements only (data not shown).

## Discussion

As an integral part of the subtelomeres, X and Y' elements have in general been thought of as heterochromatin regions with high nucleosome density [12]. We here demonstrate that this view is an oversimplification and that the subtelomeric regions of the *S. cerevisiae* genome have a distinct, chromosome specific organization, which is dependent on the precise location of X and Y' elements. These differences in chromatin structure may contribute to the formation of unique telomere structures, which will enable cellular processes to distinguish between different chromosome ends. The X elements do not interact with core histones and are devoid of nucleosomes. Instead of nucleosomes, we here demonstrate that X elements are bound by Sir3 and Rap1. In support of this finding, X elements have a strong silencing effect on adjacent genes, which are derepressed upon loss of Sir proteins or Rap1, lending functional evidence for a role of these proteins in X element chromatin architecture [6].

The MNase digest generated specific, but somewhat blurry bands, which could suggest that the specific translational positions in the subtelomeric region of chromosome V is more relaxed than what has been observed at promoters [24]. The MNase digestion therefore argues against stably positioned nucleosomes in the X-element region. In combination with ChIP analysis showing the absence of Histone H3 and the presence of Sir3, our data suggest that X-elements lack a defined nucleosomal structure.

In contrast to the X elements, the Y' elements display high histone occupancy and contains positioned nucleosomes. Moreover, H4K16Ac, which is associated with transcriptional activation and the maintenance of euchromatin, cannot be observed on X elements, but is present on Y' elements. In keeping with an active chromatin conformation, we could not observe Sir3 and Rap1 binding to Y' elements. Finally, Y' elements are actively transcribed, whereas X elements are silent. These findings support the idea that Y' elements possess anti-silencing properties and limits the spreading of silent chromatin. In X-Y' chromosome ends, the Y' element is located between the TG repeats and the X element. In spite of this, genes located near the Y' element are not silenced and a foreign reporter genes inserted in a Y' element can still be transcribed [6]. Together, these data suggest that transcription silencing does not depend on the exact distance to the chromosome end, but vary depending on the exact chromosome context. The distinct chromatin structure of Y' elements also supports the idea that these repeated regions originated as mobile elements [3]. A role for mobile genetic elements in telomere maintenance has been demonstrated in other eukaryotes. Instead of the simple telomeric repeats, *Drosophila* uses retrotransposons to elongate its chromosome ends [25].

It has been proposed that yeast telomeres form fold back loops and that this process is dependent on the Sir proteins [26]. A looping back model have been suggested previously to explain the repression patterns observed at native telomeres[6]. The finding of Sir3 and Rap1 proteins at X elements located at a distance from the TG_1–3_ repeats could therefore indicate that they play a role in this loop formation. We would like to propose that the Rap1 and Sir proteins interacting with TG_1–3_ repeats, fold back and contact the Rap1/Sir protein structure on X elements. We will address this interesting possibility in future work.

## Materials and Methods

### Yeast strains and Cell culture

Strains used for this study were BY4741 (*MATa; his3Δ1; leu2Δ0; met15Δ0; ura3Δ0*) and Y07110 (BY4741; *YLR442c::kanMX4*), obtained from EUROSCARF. Yeast cells were cultured in YPD medium containing 10 g yeast extract, 20 g peptone and 20 g glucose per liter of distilled water.

### Chromatin immunoprecipitation

ChIP assays were performed as described previously [27]. Briefly, we cultured yeast cells to a OD595 of 0.6 to 0.8, then crosslinked in 1% formaldehyde for 10 min at room temperature, quenched with 125 mM glycine, and spun down at 4°C for 5 min at 3000 rpm. Pellets were washed twice in PBS and resuspended in 400 µl cold lysis buffer with protease inhibitor cocktail (Roche). The fixed cells were lysed with 500 µl acid washed glass beads (sigma) in a FastPrep machine (FP120, BIO101 Savant) using the following program: 5 cycles,, 6.5 m/s, 20 seconds on and 1minute off. The extracts were sonicated to chromatin fragments (Bioruptor 200, Diagenode) with 5 cycles (30 seconds on, 1 minute off) at high power output. To confirm that heterochromatin is fragmented by sonication with the same efficiency as eurochromatin, we carried out southern blot analyses with 3 different probes, directed towards X elements (X1), Y' elements (Y1), and the coding region of TEF1 as an example of a euchromatin region (TEF1) (Supplementary figure S4). Southern blot results showed that the length of most sonicated fragments is around 500–1000 bp. The X and Y' elements displayed exactly the same size distribution as euchromatin. For core histone H3 and H4, chromatin was immunoprecipitated with antibodies specific for the core histone C-termini (ab1791, ab2423, and ab61240 from Abcam; and 05-858 from Upstate biotechnology Inc.) coupled to protein A beads (Sigma). For ChIP analyzes of Sir3 occupancy, we used antibodies kindly provided Dr. Hiten Madhani and Dr. Fred van Leeuwen. After wash and elution, samples were treated with proteinase K and the crosslinks were reversed overnight at 65°C. DNA was purified by phenol/chloroform extraction and ethanol precipitation, followed by incubation with RNaseA. The purified DNA was used for QPCR analyses.

### Mononucleosome preparation

BY4741 cells (1 L) were grown to OD 0.9–1.0. Then the cells were crosslinked in 1% formaldehyde for 10 min, quenched with 125 mM glycine for 5 min, and spun down at 4°C for 5 min at 3000 rpm. Pellets were washed twice in PBS and resuspended in 5 ml ice-cold lysis buffer (1 M sorbitol, 50 mM Tris-Cl [pH 7.4], 10 mM β-mercaptoethanol, and 0.5 mg/ml zymolyase). The resuspended cells were incubated at 30°C, 100 rpm shaking, in 50-ml conical tubes, to digest cell walls. After 30 minutes 10 ul cells were added to 1 ml 1% SDS solution to measure the OD value. The digestion was continued until an 80% decrease of the OD value was observed compared to the cells without zymolyase digestion. Spheroplasts were then spun down at 1,500 RPM for 10 min at 4°C. The pellets were resuspended in 4 ml digestion buffer (0.5 mM spermidine, 1 mM β-mercaptoethanol, 0.075% NP-40, 50 mM NaCl, 10 mM Tris-Cl [pH 7.4], 5 mM MgCl_2_, 1 mM CaCl_2_, and 25 units/ml Micrococcal nuclease (Sigma)) and incubated at 37°C for 20 min. To obtain mononucleosomes containing DNA with an approximate length of 146 bp, the amount of nuclease needed, was determined experimentally. The digestion was halted by shifting the reactions to 4°C and addition of EDTA to a final concentration of 10 mM. Proteinase K (final conc. 50 ug/ml) and SDS (final conc. 1%) were added to the digested material, followed by incubation at 55°C over night to reverse the crosslinking. DNA was purified by phenol/chloroform extraction followed by ethanol precipitation, and incubation with RNase A. Purified DNA was run in a 1.5% agarose gel, and 150-bp mononucleosomal DNA were extracted from the gel and used for Affymetrix tiling microarray analysis. Naked genomic control DNA was randomly digested by micrococcal nuclease and used as a genomic input control. After zymolase digestion, the spheroplasts were incubated with proteinase K (final conc. 50 ug/ml) and SDS (final conc. 1%) at 55°C over night to reverse the crosslinking and remove the nucleosome structure. DNA was purified by phenol/chloroform extraction followed by ethanol precipitation. Purified naked DNA was completely dissolved in digestion buffer and incubated at 37°C for 20 min. The digestion was stopped by EDTA (final concentration 10 mM). DNA was purified by phenol/chloroform extraction again followed by ethanol precipitation, and incubation with RNase A. Purified DNA was run in a 1.5% agarose gel. The naked DNA digested with micrococcal nuclease showed a smear around 100–500 bp on the gel. The smear around 150 bp (from 100 to 200 bp) were extracted from the gel and used for Affymetrix tiling analysis.

### Micrococcal nuclease Mapping

The Spheroplasts was prepared as described above for Mononucleosome preparations, but without formaldehyde crosslinking. Briefly, the 1 L BY4741 cells were grown to OD 0.9–1.0. Yeast pellets were washed twice in PBS and resuspended in 5 ml sorbitol buffer (1 M sorbitol, 50 mM Tris-Cl [pH 7.4], 10 mM β-mercaptoethanol, and 0.5 mg/ml zymolyase). After we reached an 80% decrease in the OD value, spheroplasts were spun down at 1,500 RPM for 10 min at 4°C. The pellets were resuspended in 4 ml digestion buffer (0.5 mM spermidine, 1 mM β-mercaptoethanol, 0.075% NP-40, 50 mM NaCl, 10 mM Tris-Cl [pH 7.4], 5 mM MgCl_2_, 1 mM CaCl_2_, and different amounts of Micrococcal nuclease (Sigma)). The amounts of micrococcal nuclease used are indicated in the figure legend and the incubation was at 37°C for 10 min. EDTA (final concentration of 10 mM), Proteinase K (final conc. 50 ug/ml) and SDS (final conc. 1%) were used to stop the digestion. The samples were incubated at 55°C for two hours. DNA was purified by phenol/chloroform extraction, then incubated at 37°C for 30 min, purified again by phenol/chloroform again and precipitated by ethanol. Purified DNA was digested by 50 units of ScaI overnight at 37°C. The digested DNA was separated in an 1.4% agarose gel, 150 volt, 4 hours. The DNA was transferred onto a Hyrbid N+ membrane and fragments were detected by Southern blotting. The probe was amplified by PCR using a primer pair (MR5F:TTC ATC TTC TGA CGC GGT GAG CTT MR5R:TCA AGT CCA TTG GCA GCA CCT TTG). The purified PCR product was labeled by the Stratagene random primer labeling kit. Hybridization was performed at 65°C in rapid-hyb buffer (GE healthcare). The Naked DNA control preparation was the same as we described for preparation of mononucleosomes. The Spheroplasts were incubated with proteinase K to remove all proteins. The naked genomic DNA was purified by phenol/chloroform extraction, and ethanol precipitation. The precipitated DNA was dissolved in lysis buffer and amounts of micrococcal nuclease used, are indicated in the figure legend. After 10 minutes of nuclease digestion, the reactions were stopped by addition of EDTA and SDS followed by phenol/chloroform purification of DNA. Precipitated DNA was dissolved in water and digested with 50 units of *Sca* I overnight. The digested naked DNA was run in parallel with micrococcal nuclease treated samples in an agarose gel and analyzed as described [28].

### Tiling microarray, labeling, and hybridization

For *S. cerevisiae* tiling array analyzes, labeling and hybridization were performed according to the manual from Affymetrix. Five ug mononucleosomes and naked genomic DNA control were labeled by biotin following the Affymetrix labeling kit protocol and hybridized in parallel to tiling arrays. After hybridization, microarrays were scanned with the Scanner 3000 and GeneChip Operating Software (Affymetrix). All labeling and hybridization were carried out at the Bioinformatics and Expression Analysis Core Facility at Karolinska Institutet, Stockholm. All tilling array experiments were performed with two independent biological repeats.

### Microarray Data analysis

Microarray data sets were analyzed using the Affymetrix tiling array software (TAS). Genome annotation data were obtained from the Affymetrix website. Mononucleosome data were selected as the treatment group. Random digested input control data were selected as the background group. Bandwidth was 70 and the test type was two sides. After intensity analyses, the log2 transformed signal file was visualized by the integrated genome browser (Affymetrix). Raw data and signal files obtained from TAS have been deposited on the NCBI GEO website (GSE13615).

### Real-time PCR

Real-time quantitative PCR was carried out on a Bio-Rad CFX-96 detection system with QPCR SYBR Green reagents (Bio-Rad) and with a primer concentration of 0.5 µM. PCR conditions were standardized to 40 cycles; 95°C for 10 s, 55°C for 10 s, and 72°C for 30 s. Results were analyzed as described. Serial dilutions of total extract DNA (1/10,000, 1/1000, 1/100, and 1/10) were used to generate a standard curve for each primer and each reaction. Each ChIP experiment was normalized to total DNA input. The *TEF1* (euchromatin) and *HMR* (hetrochromatin) genes were used as controls. Experiments were typically repeated at least 3 times, error bars in figures show standard deviations. Primer sequences are presented in supplementary table S1.

### Reverse-Transcription PCR

Yeast total RNA was extracted using the RNeasy Mini Kit (Qiagen) following the manufacturers instructions. Briefly, 2×10^7^ yeast cells were harvested by centrifugation, the pellet was resuspended in sorbitol buffer (1 M sorbitol, 0.1 M EDTA PH7.4, and 50 U zymolase) and incubated for 30 minutes at 30°C. The spheroplasts were collected by centrifugations for 5 min at 1500 rpm. Lysis buffer was added and the spheroblasts were lysed by vortexing vigorously. One volume 70% ethanol was added to the homogenized lysate and mixed well by pipetting. The sample was transferred to an RNeasy column and spun down for 15 seconds at 10,000 RPM. After washing, the RNA was eluted with 50 µl elution buffer. The RNA concentration was determined using nanodrop spectrophotometer (Thermo Scientific). One ug RNA was used for first strand cDNA synthesis (Roche). Quantitative PCR was carried out on a Bio-Rad CFX-96 detection system with QPCR SYBR Green reagents (Bio-Rad) and with a primer concentration of 0.5 µM. PCR conditions were standardized to 25°C 10 minutes, 95°C 3 minutes and 40 cycles; 95°C for 10 s, 60°C for 30 s. A set of 4 genes with known mRNA abundance YHR021(8200), YDR418W(4200), YGL245W(980), and YDL218W(124) were used as abundance standards. cDNA prepared from BY4741 and a *sir3* mutant strain were used for transcription analysis. Data analysis was performed with Bio-Rad CFX Manager Software. Different samples were normalized to ACT1, which was used as an internal control. Target gene primer efficiency was normalized by genomic DNA serial dilution controls. Target gene mRNA abundance was calculated based on comparison with genes with known mRNA abundance. All reported experiments represent at least three biological repeats.

## Supporting Information

Figure S1 Nucleosome density. High resolution profiling of nucleosome position of X and Y elements. X elements presented by red rectangle and Y elements presented by Yellow rectangle. The black arrow indicated the X element nucleosome free regions. The width of these regions showed above the black arrow. X axis scale vary between different telomeres due to different length of Y elements.
(2.58 MB PDF)

Figure S2 Rap1 binding. Rap1 occupancy showed high enrichment to X element. The original data were obtained from lieb JD et al,2001 Nat Genet. X and Y elements indicated as red and yellow rectangle.
(0.28 MB PDF)

Figure S3 H4K16 Acetylation. High resolution profiling of H4K16Ac of X and Y elements. H4K16 Ac anbitbody(abcam) enriched DNA and input DNA hybrid to tiling array respectively. H4K16ac IP data were normalized to input data in TAS and visualized in IGB as we described in methods. X and Y element indicated as the same as that in Figure 1.
(2.28 MB PDF)

Figure S4 Southern blotting of X,Y and TEF1 probe. Southern blot showed the fragment size of input. Probes used here were X element(X1), Y element(Y1) and euchromatin control(TEF1). Marker lane indicated the molecular weight.
(0.45 MB PDF)

Table S1 The primer sequences used in this study.
(0.62 MB PDF)
