# Supplementary material for: Distinct Differences in Chromatin Structure at Subtelomeric X and Y' Elements in Budding Yeast
Source: PLoS One. 2009 Jul 23;4(7):e6363. doi: 10.1371/journal.pone.0006363 (PMC2709909; doi:10.1371/journal.pone.0006363)

Supplementary information figure S1 A.Nucleosome density

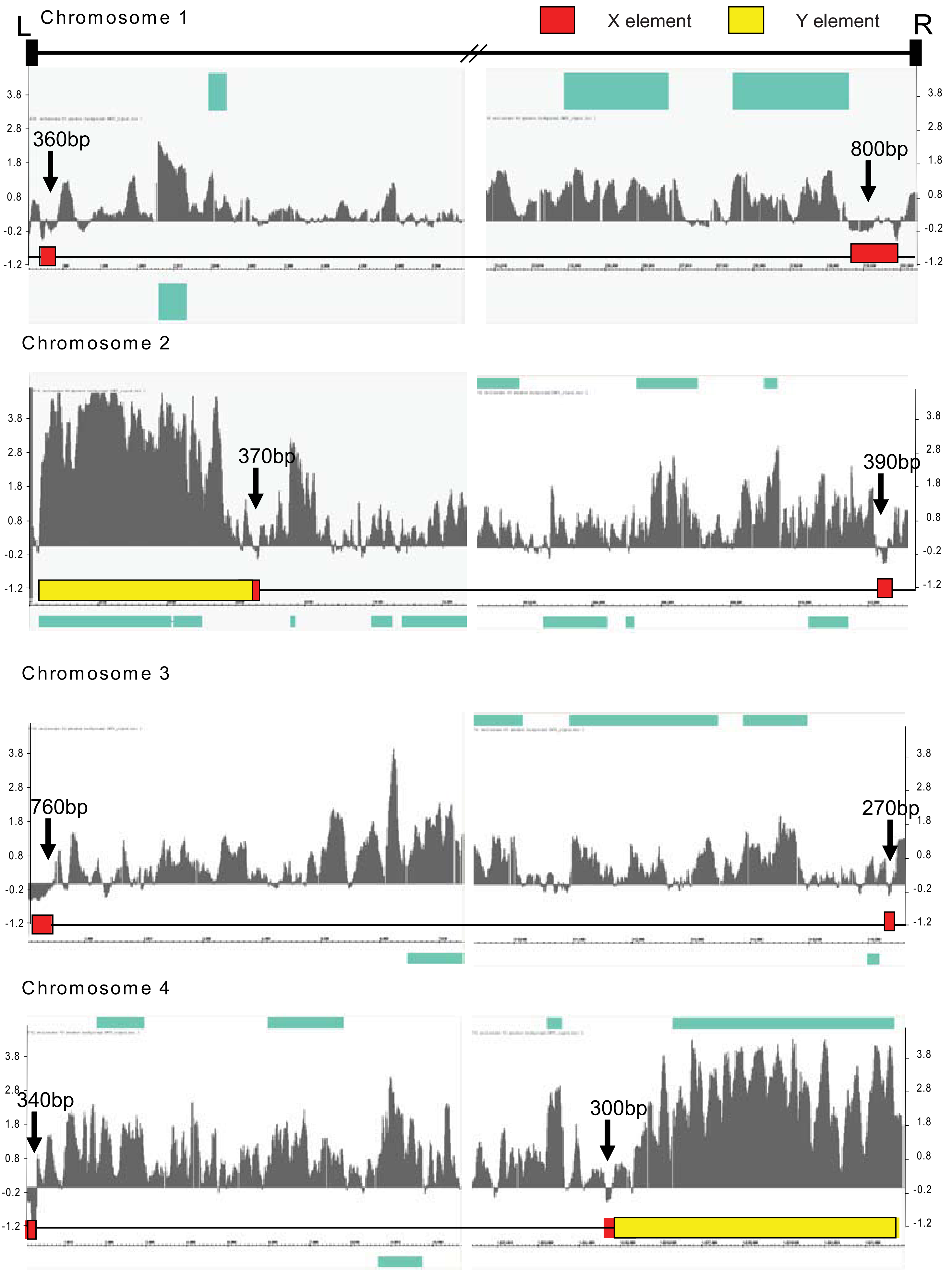

Supplementary information figure S1 B.Nucleosome density

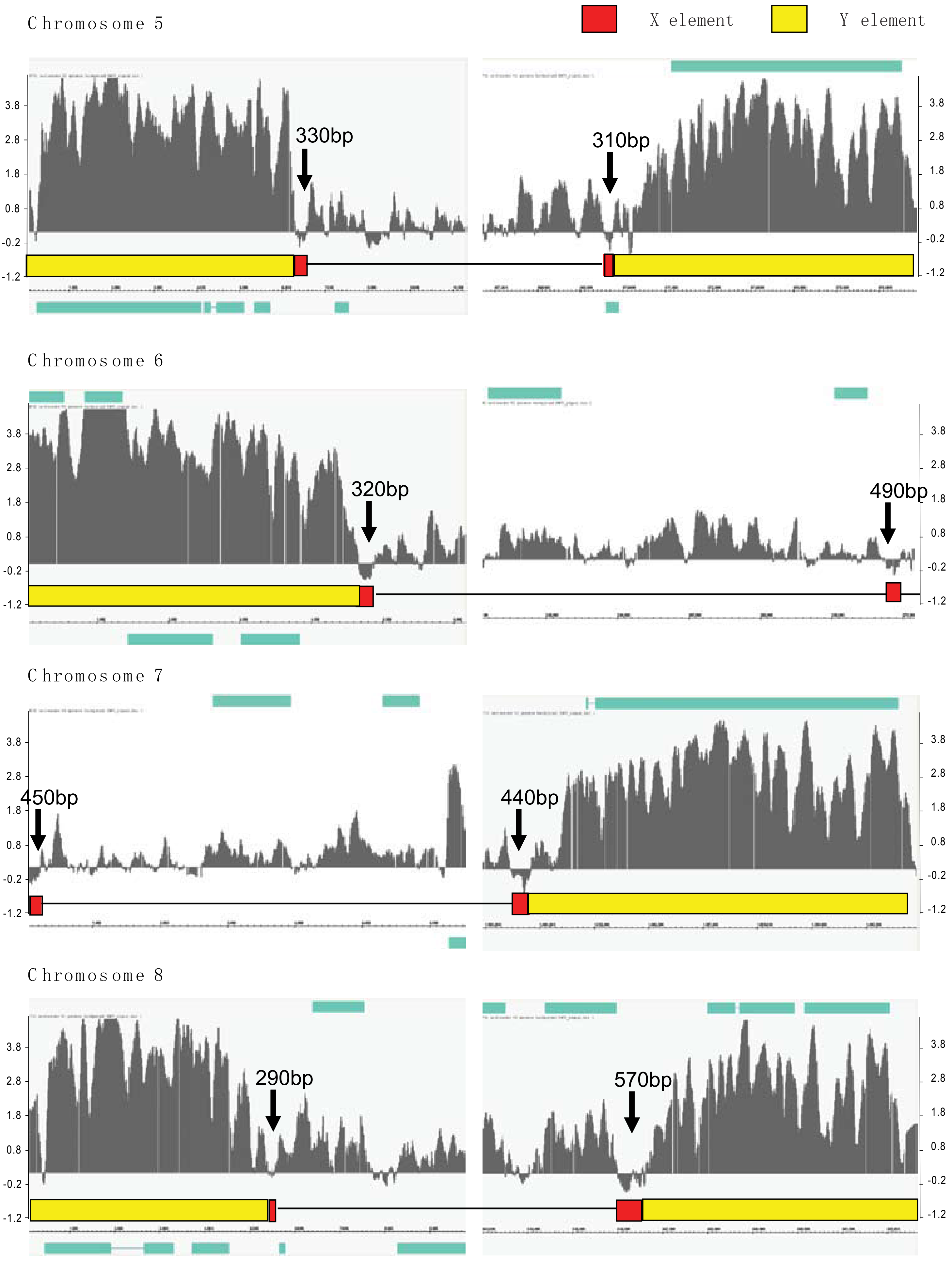

Supplementary information figure S1 C.Nucleosome density

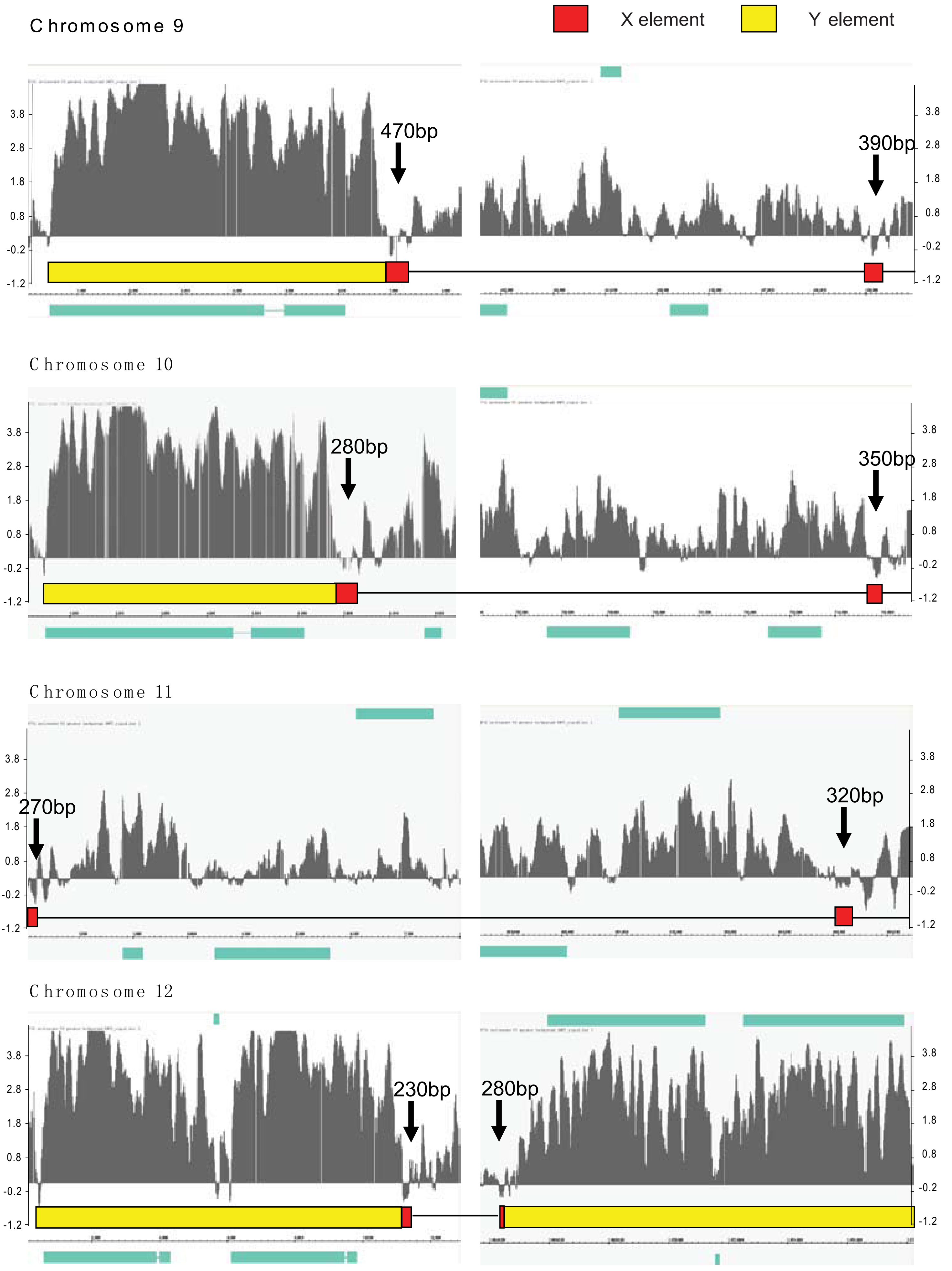

Supplementary information figure S1 D.Nucleosome density

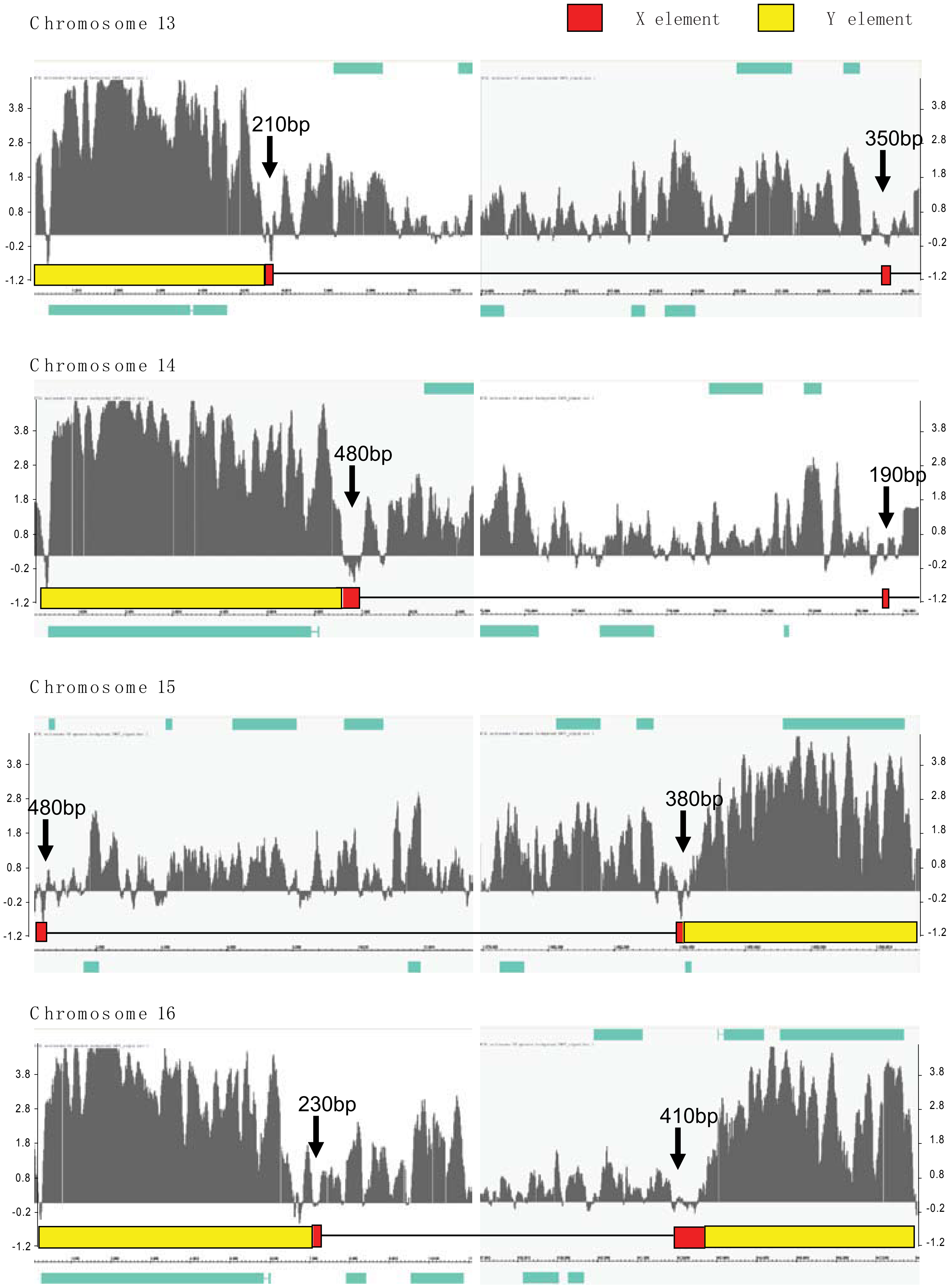

Supplement: Figure S1 — Nucleosome density. High resolution profiling of nucleosome position of X and Y elements. X elements presented by red rectangle and Y elements presented by Yellow rectangle. The black arrow indicated the X element nucleosome free regions. The width of these regions showed above the black arrow. X axis scale vary between different telomeres due to different length of Y elements. (2.58 MB PDF) [file pone.0006363.s001.pdf]
