# Supplementary material for: Distinct Differences in Chromatin Structure at Subtelomeric X and Y' Elements in Budding Yeast
Source: PLoS One. 2009 Jul 23;4(7):e6363. doi: 10.1371/journal.pone.0006363 (PMC2709909; doi:10.1371/journal.pone.0006363)

Supplementary information figure S2 A. Rap1 binding.

Data from: Lieb JD, Liu X, Botstein D, Brown PO.
Nat Genet. 2001 Aug;28(4):327-34.

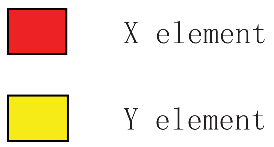

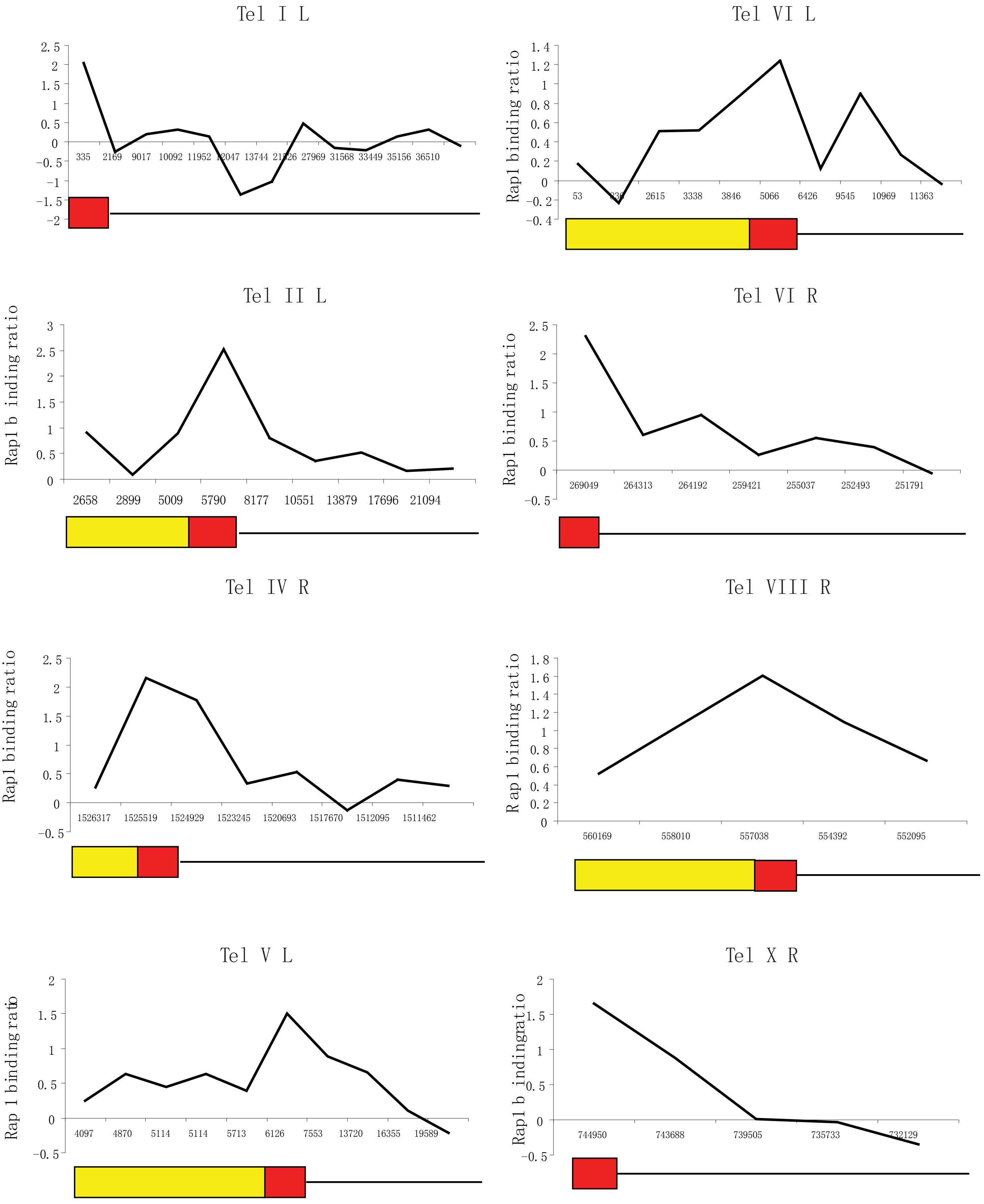

Supplementary information figure S2 B. Rap1 binding.

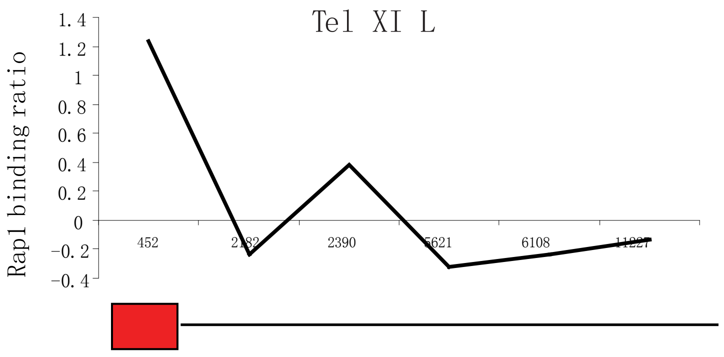

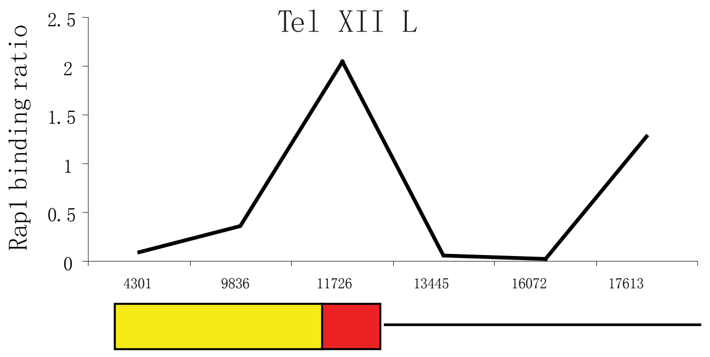

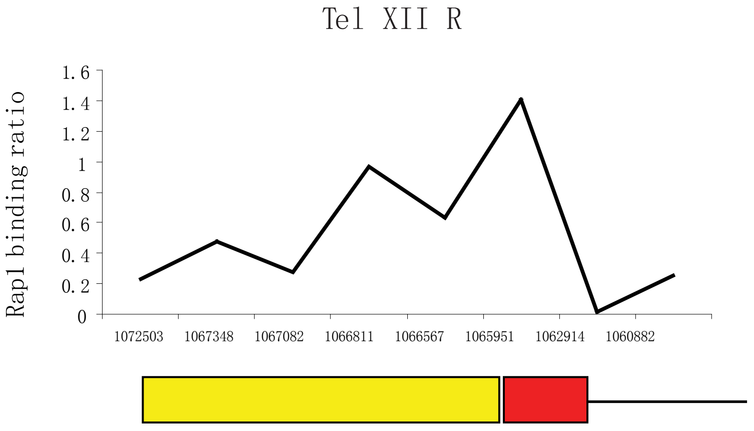

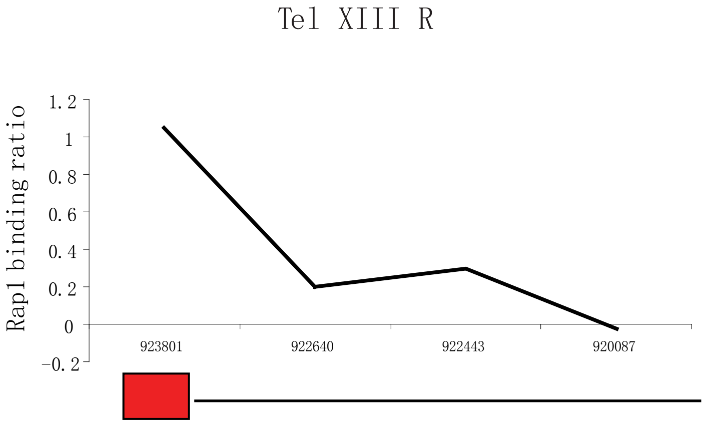

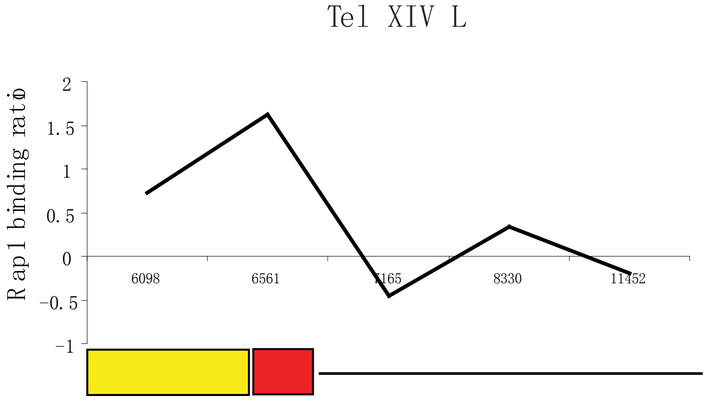

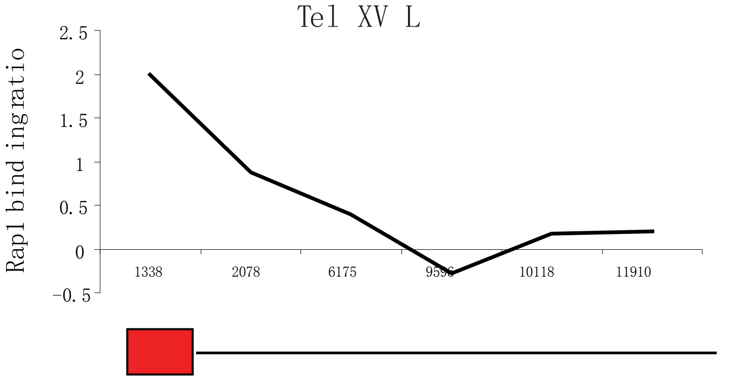

Supplement: Figure S2 — Rap1 binding. Rap1 occupancy showed high enrichment to X element. The original data were obtained from lieb JD et al,2001 Nat Genet. X and Y elements indicated as red and yellow rectangle. (0.28 MB PDF) [file pone.0006363.s002.pdf]
