# Supplementary material for: Distinct Differences in Chromatin Structure at Subtelomeric X and Y' Elements in Budding Yeast
Source: PLoS One. 2009 Jul 23;4(7):e6363. doi: 10.1371/journal.pone.0006363 (PMC2709909; doi:10.1371/journal.pone.0006363)

Supplementary information figure S3 A.H4K16 Acetylation

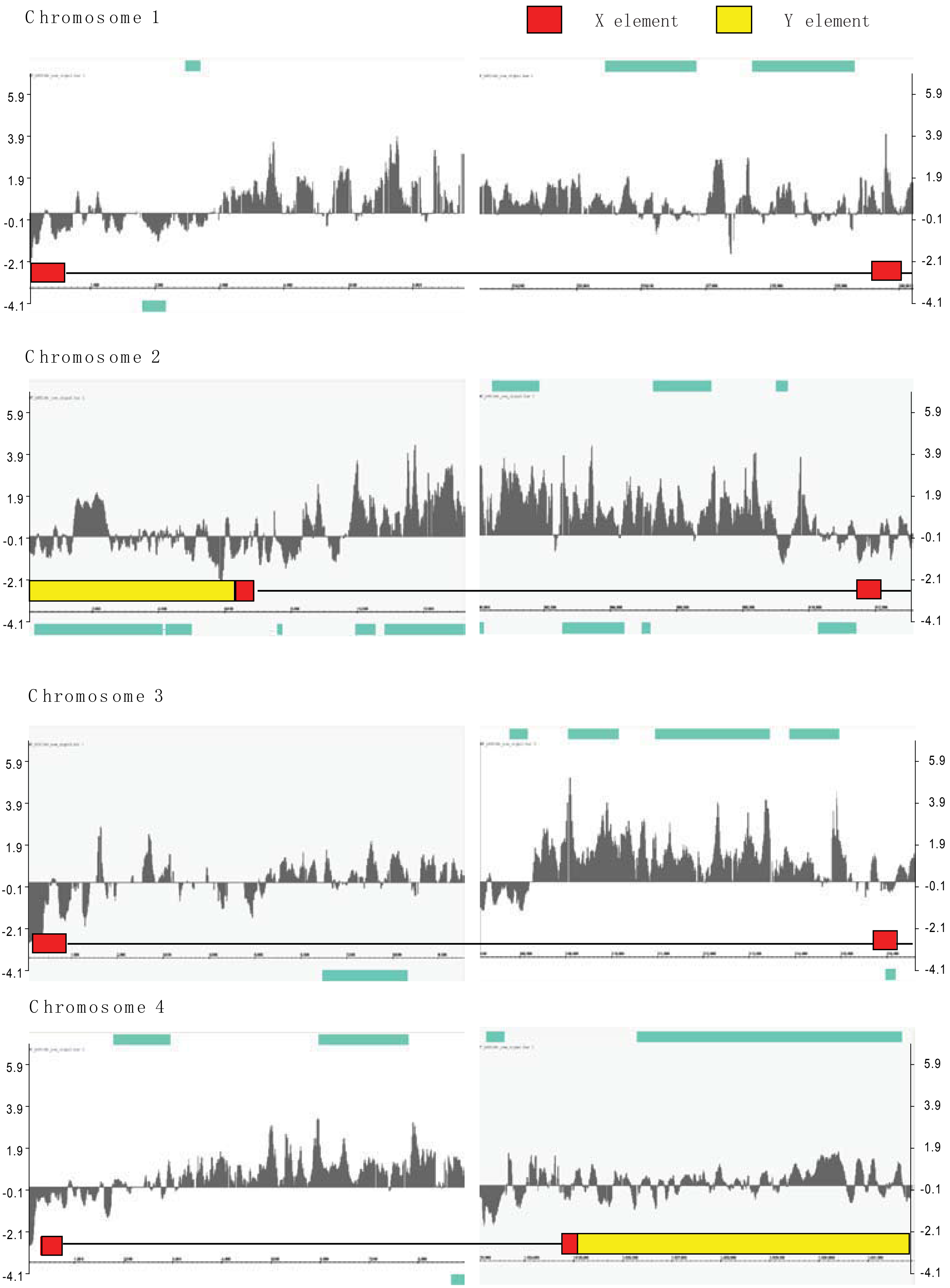

Supplementary information figure S3 B.H4K16 Acetylation

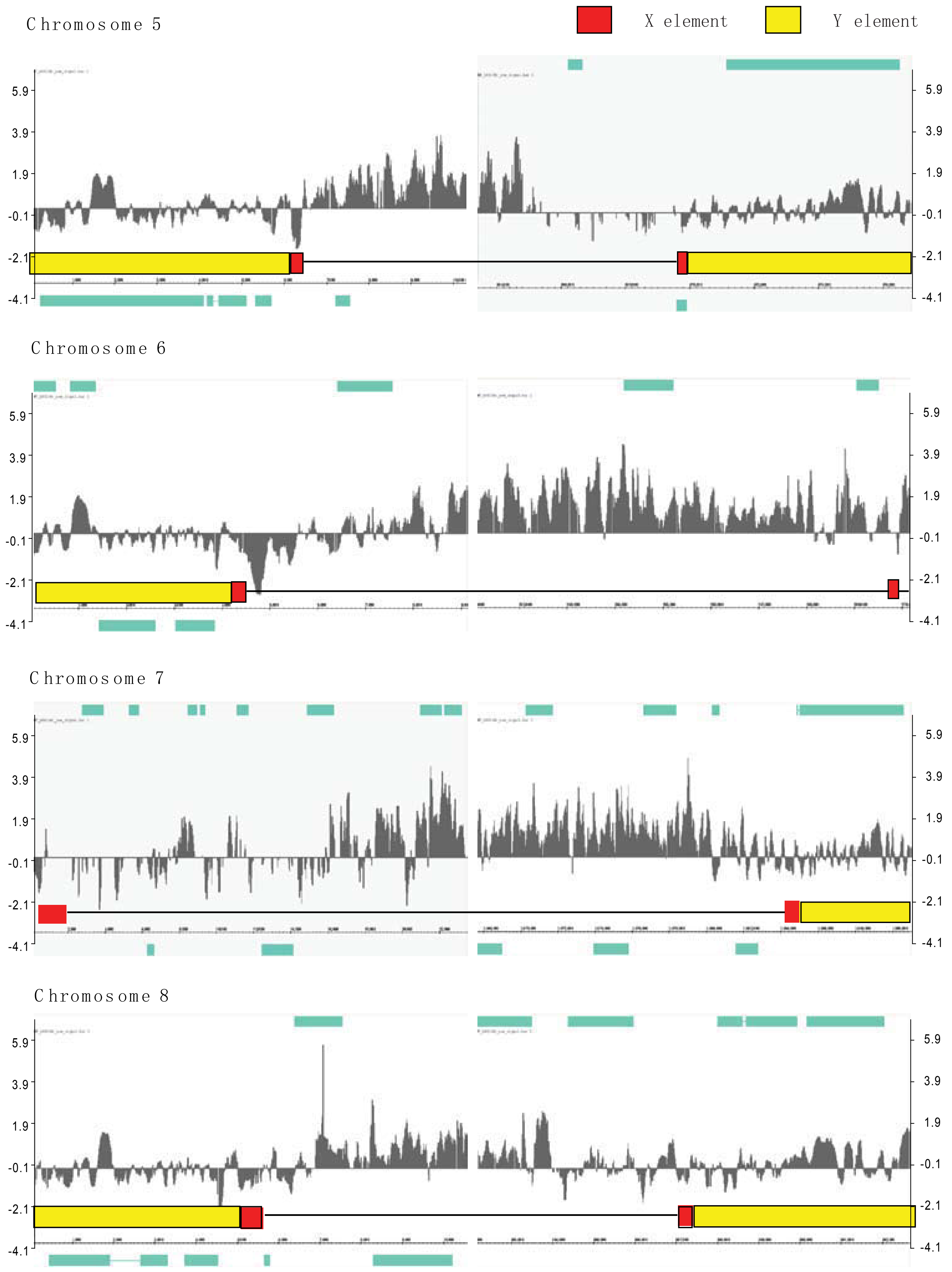

Supplementary information figure S3 C.H4K16 Acetylation

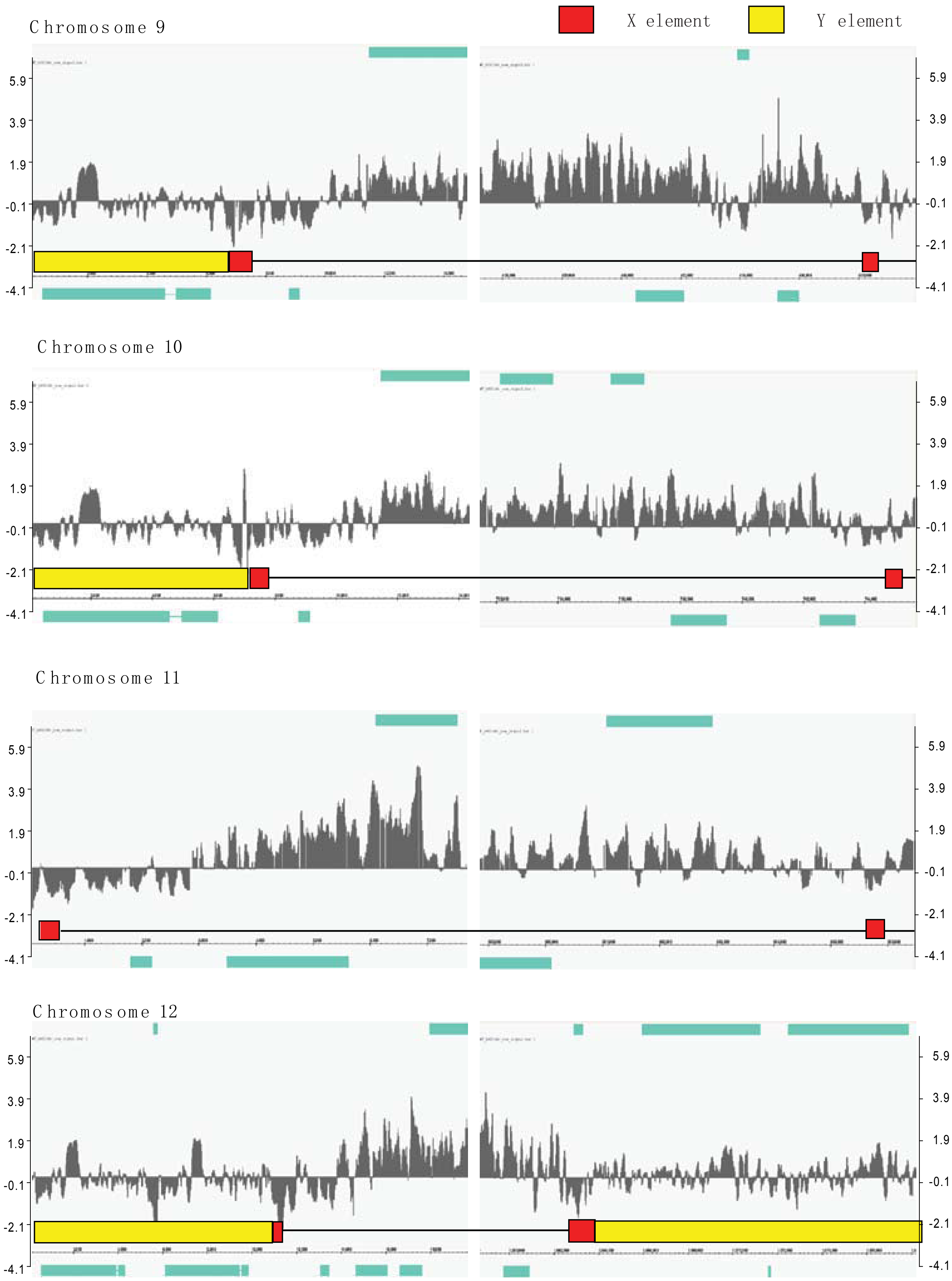

Supplementary information figure S3 D.H4K16 Acetylation

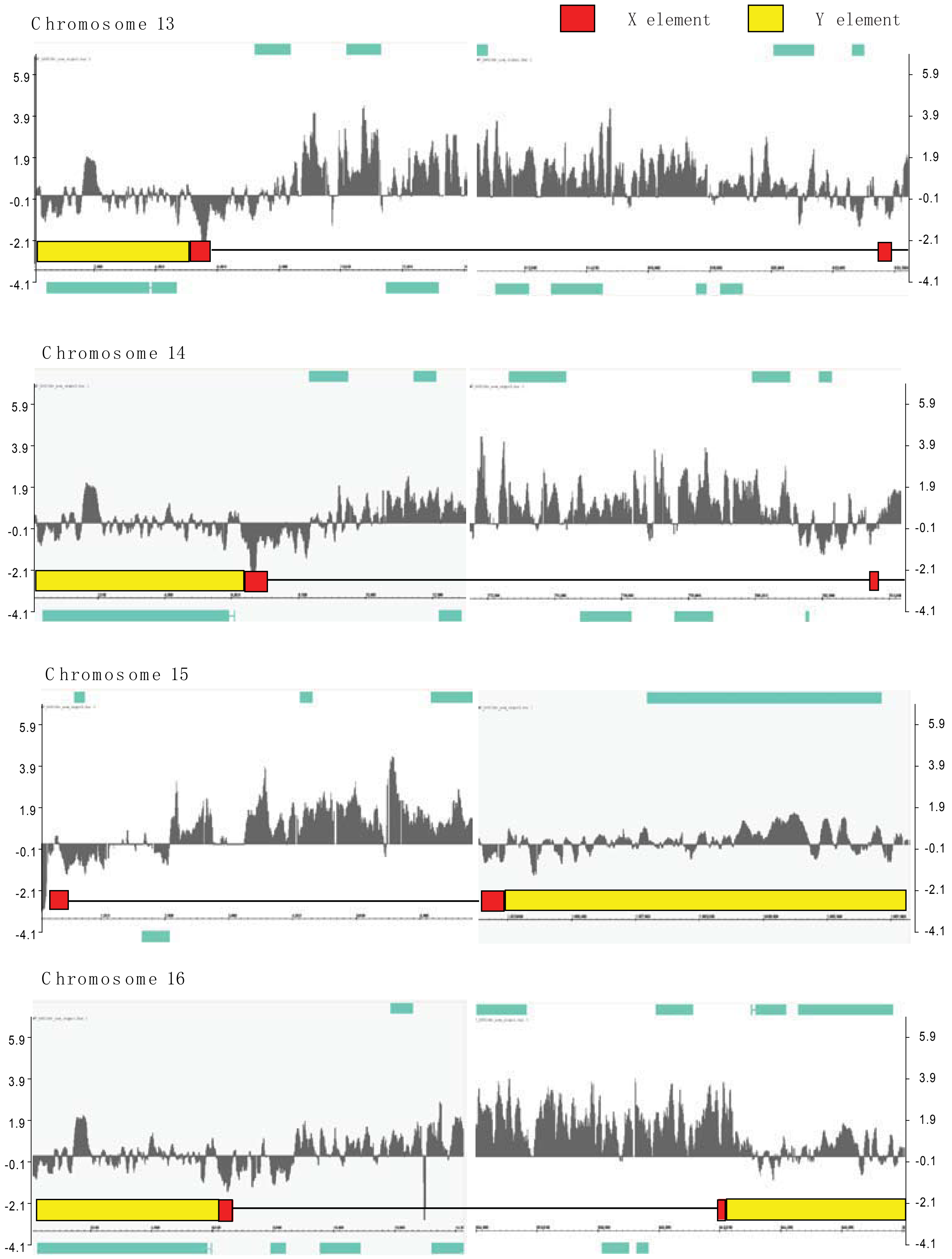

Supplement: Figure S3 — H4K16 Acetylation. High resolution profiling of H4K16Ac of X and Y elements. H4K16 Ac anbitbody(abcam) enriched DNA and input DNA hybrid to tiling array respectively. H4K16ac IP data were normalized to input data in TAS and visualized in IGB as we described in methods. X and Y element indicated as the same as that in Figure 1. (2.28 MB PDF) [file pone.0006363.s003.pdf]
