# Supplementary figures and images for: Distinct Differences in Chromatin Structure at Subtelomeric X and Y' Elements in Budding Yeast
Source: PLoS One. 2009 Jul 23;4(7):e6363. doi: 10.1371/journal.pone.0006363 (PMC2709909; doi:10.1371/journal.pone.0006363)

supplymentory information Figure S4

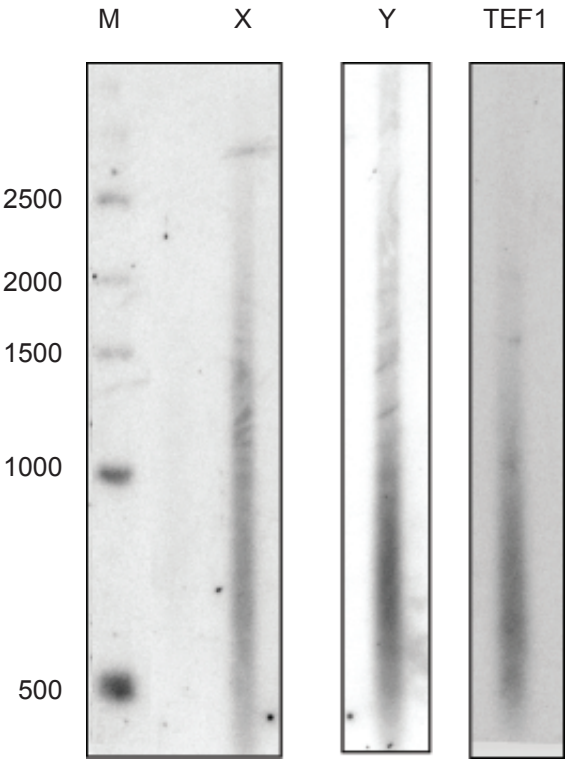

Supplement: Figure S4 — Southern blotting of X,Y and TEF1 probe. Southern blot showed the fragment size of input. Probes used here were X element(X1), Y element(Y1) and euchromatin control(TEF1). Marker lane indicated the molecular weight. (0.45 MB PDF) [file pone.0006363.s004.pdf]
