# Supplementary material for: Distinct Differences in Chromatin Structure at Subtelomeric X and Y' Elements in Budding Yeast
Source: PLoS One. 2009 Jul 23;4(7):e6363. doi: 10.1371/journal.pone.0006363 (PMC2709909; doi:10.1371/journal.pone.0006363)
Supplement: Table S1 — The primer sequences used in this study. (0.62 MB PDF) [file pone.0006363.s005.pdf]

Table S1. All primer pairs used in the study

Histone density primer:

C1RF	TGAGGTAAGTGCCGTGGATTGTGA
C1RR	ACACCCACACACCACACCCTAATA
P1F	ACACACGTGCTTACCCTACCACTT
P1R	TGCCGTGCATAATGATGTGAGTGC
P2F	ACGGCATTCCTGTCGATGCTGATA
P2R	CAGCGAGAACTTCAACGTTTGCCA
P3F	TATTTGGGCCCGCCGAATGAGATA
P3R	ATGCACCCACATCATTATGCACGG
P4F	GCTGAGGCAAGTGCCGTGAATAAT
P4R	TGTCATACGCACACGGATGCTACA
TEF1F	AATCTCTGGTTGGAACGGTGAC
TEF1R	CTCAATGGCTTGTCAGTTGGTC

Tel 7 L primers

Tel7L0.5kF	GCTGAGGCAAGTGCCGTGAATAAT
Tel7L0.5kR	ACTACCATCCACCGCCCATCATAA
Tel7L1.5kF	TGTCCAGCGTTGGTAAACAGTTGC
Tel7L1.5kR	ACAGCCCGAGCCTGTACTAAATGT
TEL7L4KF	CCAACTGACAAAGACGCTTGCGAA
TEL7L4KR	TGGTCTCAAATTGCGAAGAGGACG
TEL7L6.5KF	ATACTACATGTTCCAAGCCGCCCA
TEL7L6.5KR	ACATGGTGGTGAAGTCACCGTAGT
TEL7L9KF	ACGTCCGTACCATAGGTTCCTTCT
TEL7L9KR	ACATTCACTGGGCCTCGTTCTACT
TEL7L11.5KF	TCAGCAGTGTCTGCCCAAGAGTTA
TEL7L11.5KR	TCCATTGGTGACGCTATGGTTCCT
TEL7L14KF	AATTGCTTGCAGCAGGTTGATGGG
TEL7L14KR	TAGACGCATGGTAAACCTCGCCAT
TEL7L 16.5KF	AGGCACACGCATAATTGACG
TEL7L 16.5KR	GCCTGCCATTTGACTAACCCAGTT
TEL7L 19KF	AGATGAAACGACTTGCTGTGCTGC
TEL7L 19KR	AACTTCACGCCCACGTTGAGAGTA

X-Y elements primers

Y1 0.8KF	ACGGCATTCCTGTCGATGCTGATA
Y1 0.8KR	ATGTGGTAACAACCACACCTCCGA
Y2 1.8KF	TGCTAGCACCAACTCCAGCACTAA
Y2 1.8KR	TTATTGGCGTCCTCCTTGGCACTA
Y3 3.0KF	TCAAACGGTCGGAAGATCTCAGCA
Y3 3.0KR	AAGGCACCTCGGATTTCTCCTTGA
X 6.0K F	TATTTGGGCCCGCCGAATGAGATA
X 6.0K R	AGCGGTCTATACCCTGTGCCATTT
IMD27.3KF	CCAGGGAACAAGCTGCCAATTTGA
IMD27.3KR	TGTGGCCTACCACAAGCCATAACT

transcription abundance standard primers

YHR021CF	GATTTGTTGCACCCAACTGCTGCT
YHR021CR	TGGTGATGTTCAAACAACCTGGGC

YDR418WF	GCCAAGGCCACCAAGGAATTCAAA
YDR418WR	GCAGATGGAACAACAGAAGCAGCA
YGL245WF	ACGATCCAAGGTTCCCAACTGTCA
YGL245WR	ACTGGGTTGACGATAGCAGTGTGT
YDL218WF	GCATGTAATTCTTCTCAGGCGGCA
YDL218WR	CGCTTCTCCACATTCCTTGGGTTT
Act1F	ACGTTCCAGCCTTCTACGTTTCCA
Act1R	ACGTGAGTAACACCATCACCGGAA

Transcription primers

YRF1-1F	AGGCCGGAAATCAAGCGATGAATG
YRF1-1R	GCAAATCTCCAGCAGCAGCAAAGA
FLO1F	AGAGCTCTGCAGCAGGGTTAATCT
FLO1R	CCGGAAGAGAGCTTACATCAACGA
YLL067cF	TGAAGACGAACCAGATTTCCAGGG
YLL067cR	AAGTACGCATCGTAGAGCTGTCGT
YHR219WF	TGCTAGCACCAACTCCAGCACTAA
YHR219WR	TTATTGGCGTCCTCCTTGGCACTA
YHR218WF	TCAAACGGTCGGAAGATCTCAGCA
YHR218WR	AAGGCACCTCGGATTTCTCCTTGA
YHR217F	TATTTGGGCCCGCCGAATGAGATA
YHR217R	AGCGGTCTATACCCTGTGCCATTT
YFL063F	GCATACGCACACGGATGCTACAAT
YFL063R	CCGTGCATAATGATGTGGGTGCAT

For sir3 occupancy primer

HMR-EF	CCCGTCCAAGTTATGAGCTTAATC
HMR-ER	TTTTTCGGAATCGAGAATCTTCGT

Note:
P1 amplified C1L,5R and C13R;
P2 amplified a subset of Y' elements in C2L, C4R, C7R, ,C8L&R, C9L, C12L&R, C13L, C14L, C15R, and C16L&R.
P3 amplified C2L, C3R, C4L, C5R, C7R, C8L, C9L, C10L&R, C11R and C14R.
P4 amplified C3L, C4L&R, C5R, C7L, C9L, C10L, C11R, C12R, C13L&R and C15R.
